# Anterior Segment Optical Coherence Tomography Angiography: A Review of Applications for the Cornea and Ocular Surface

**DOI:** 10.3390/medicina60101597

**Published:** 2024-09-28

**Authors:** Brian Juin Hsien Lee, Kai Yuan Tey, Ezekiel Ze Ken Cheong, Qiu Ying Wong, Chloe Si Qi Chua, Marcus Ang

**Affiliations:** 1 Singapore National Eye Centre, Singapore 168751, Singapore; brian.lee@mohh.com.sg (B.J.H.L.); teykaiyuan@gmail.com (K.Y.T.); wong.qiu.ying@seri.com.sg (Q.Y.W.); chloe.chua.s.q@seri.com.sg (C.S.Q.C.); 2 Singapore Eye Research Institute, Singapore 168751, Singapore; 3 Ophthalmology and Visual Sciences Academic Clinical Program, Duke-NUS Medical School, Singapore 169857, Singapore; ezekiel@u.duke.nus.edu

**Keywords:** anterior segment, cornea, ocular surface, optical coherence tomography angiography

## Abstract

Dye-based angiography is the main imaging modality in evaluating the vasculature of the eye. Although most commonly used to assess retinal vasculature, it can also delineate normal and abnormal blood vessels in the anterior segment diseases—but is limited due to its invasive, time-consuming methods. Thus, anterior segment optical coherence tomography angiography (AS-OCTA) is a useful non-invasive modality capable of producing high-resolution images to evaluate the cornea and ocular surface vasculature. AS-OCTA has demonstrated the potential to detect and delineate blood vessels in the anterior segment with quality images comparable to dye-based angiography. AS-OCTA has a diverse range of applications for the cornea and ocular surface, such as objective assessment of corneal neovascularization and response to various treatments; diagnosis and evaluation of ocular surface squamous neoplasia; and evaluation of ocular surface disease including limbal stem cell deficiency and ischemia. Our review aims to summarize the new developments and clinical applications of AS-OCTA for the cornea and ocular surface.

## 1. Introduction

Optical coherence tomography (OCT) is an indispensable ocular imaging modality in our routine clinical practice [1]. It creates three-dimensional tomographic images by applying low-coherence light and measuring the echo time delay of light backscattered from tissue structures [2]. OCT is non-invasive and can provide fast, high-resolution images of the eye to assess both its anterior and posterior segments [3].

Fluorescein angiography (FA) and indocyanine green angiography (ICGA) are standard imaging modalities for evaluating the vasculature of the eye. Although most commonly used for the retina, they can also delineate normal and abnormal blood vessels in anterior segment diseases, even for those potentially obscured by corneal scarring [4]. FA and ICGA can reliably quantify various vessel parameters and maturity [5]. Contrast leakage in angiography can shed insight into vessel maturity and pathological states while differentiating between afferent and efferent vessels [6]. However, it also can obstruct the visualization of deeper vessels, resulting in an underestimation of the vascularity [7]. FA and ICGA are also limited due to their invasive, time-consuming, and subjective nature. They are also contraindicated in patients who are pregnant or have significant hepatic or renal impairments. There is also an inherent risk of adverse effects from the intravenous infusion of contrast such as nephrotoxicity and even life-threatening anaphylactic reactions [8].

OCT angiography (OCTA) is a relatively recent non-invasive imaging modality capable of producing high-resolution angiographic images of the eyes, in multiple coronal planes, within seconds [9]. It performs additional analyses of signal decorrelation between consecutive OCT scans by comparing phase speckle contrast, changes in intensities, and variations in the OCT signal [1]. OCTA produces high-resolution images with an acquisition time comparable to FA and ICGA while avoiding the risks of contrast-related complications [10]. While a clinician is required for the administration of intravenous contrast in contrast-based angiography, OCTA only requires a technician to operate the device. Currently, OCTA is utilized when imaging the vasculature of the posterior segment, such as the retina, choroid, and optic nerve, and for patients who are contraindicated for FA and ICGA [11,12]. With growing interest in anti-angiogenic therapies for anterior segment diseases, there is an increasing preference for a safe, rapid, and non-invasive method to assess the anterior segment vasculature [13]. Available anterior segment OCTA (AS-OCTA) systems for the cornea and ocular surface include AngioVue (Optovue Inc. Fremont, CA, USA), Angioscan (Nidek Co. Ltd, Gamagori, Japan), DRI OCT Triton (Topcon, Tokyo, Japan), PlexElite (Carl Zeiss Meditec, Dublin, California, USA), Angioplex (Carl Zeiss Meditec, Dublin, California, USA), Spectralis OCTA (Heidelberg Engineering, Heidelberg, Germany), and Yalkaid and BMizar (TowardPi Medical Technology Co., Ltd., Beijing, China), all of which merely require an additional adaptor lens to image the anterior segment [1,14,15]. Table 1 summarizes the advantages and disadvantages of AS-OCTA and dye-based angiography for the anterior segment.

In this review, we aim to summarize the recent developments and applications of AS-OCTA for the cornea and ocular surface, specifically the conjunctiva, episclera, and sclera, as well as discuss potential areas for further research.

## 2. Methodology

We conducted a literature review on PubMed, Web of Science, and Google Scholar with articles identified through multiple search methods. These included but were not limited to key terms such as “optical coherence tomography angiography”, “OCTA”, “anterior segment”, and “ocular surface”. Articles were included only if they were less than 5 years old and relevant to our review. A total of 138 articles were identified initially prior to selection. An overview of the recent applications of OCTA for the anterior segment, which we included in our review, is provided in Table 2. 

## 3. Vascular Anatomy of the Cornea and Ocular Surface

The cornea is a transparent connective tissue in the anterior segment that provides a structural barrier for the intraocular structures against the external environment, whilst providing two-thirds of its refractive power [33]. It consists of five different avascular layers—epithelium, Bowman layer, stroma, Descemet’s membrane, and endothelium [34]. The main source of nutrients to the cornea is derived from the aqueous humor in the anterior chamber, supplemented by diffusion via the limbal vessels [33]. The transparency of a healthy cornea is dependent on its avascularity, allowing for optimal transmission of light and refraction for visual processing [35]. Avascularity of the cornea is achieved via a delicate balance between angiogenic (vascular endothelial growth factor (VEGF), fibroblast growth factor, angiogenin, etc.) and anti-angiogenic factors (angiostatin, endostatin, matrix metalloproteinases, etc.) through the inhibition of both inflammatory and immune responses [6,36,37]. Corneal neovascularization (CoNV) is a pathological development of blood vessels in the cornea that occurs within the deep or superficial layers of the cornea. CoNV can arise from a myriad of etiologies, ranging from infections such as bacterial infectious keratitis secondary to *Pseudomonas* infection; inflammatory conditions like marginal keratitis, atopic keratoconjunctivitis, or corneal graft rejection; to chronic hypoxic states secondary to contact lens overuse, limbal stem cell deficiency (LSCD), or chemical injury to the eye [37]. CoNV can result in corneal scarring, edema, and persistent inflammation—all of which can worsen visual outcomes [38,39]. CoNV can also disrupt the immunologically privileged state of the cornea, which results in higher rates of corneal graft rejection and failure [40]. These complications of CoNV, which can eventually lead to blindness, can be avoided with early CoNV detection and intervention, whether medical or surgical [41]. The detection of ghost vessels (remnant vessels from CoNV), can also provide insight into previous ocular insults in patients.

The ocular surface includes the conjunctiva, a continuous vascular membrane extending from the palpebral portion of the eyelids at its margins to the fornix and the bulbar portion over the anterior sclera at the limit of the corneoscleral limbus [42]. It is composed of a surface layer of non-keratinizing stratified squamous epithelium with underlying vascular stroma [33]. Beneath the bulbar conjunctiva is the episclera, a thin vascular layer of connective tissue with fibers that blend with the underlying scleral stroma. The sclera is a dense and avascular fibrous layer that provides structure to the globe and protects the intraocular structures against external injury. The irregular arrangement of its connective tissue matrix contributes to its opacity, which reduces internal light scattering for optimal image processing by the retina [43]. The vasculature of the conjunctiva, episclera, and sclera consists of extensive anatomical networks of branching capillaries, arterioles, and venules. The conjunctival vessels are mainly derived from the ophthalmic artery, which includes the marginal and peripheral tarsal arcades, and the anterior and deep ciliary systems [44]. The episcleral vessels branch off from the anterior ciliary vessels and have extensive anastomoses to form the deep episcleral capillary circulation. The episcleral vessels also enter the bulbar conjunctiva at the limbus to form the anterior conjunctival arteries, communicating with branches of the posterior conjunctival arteries, and giving rise to the pericorneal plexus [44]. The episcleral venous plexus and mid and deep scleral venous plexuses are formed from the anastomoses of the ciliary venous plexus and collector channels from Schlemm’s canal [10]. The translucent appearance of the conjunctiva allows for in vivo, non-invasive visualization of the conjunctival and episcleral/sclera vasculatures and microcirculation. Pathologies of the conjunctiva and episclera/sclera often respond with vasodilation and observable hyperemia. These conditions can be infectious and non-infectious, local or systemic, including viral conjunctivitis, episcleritis, scleritis, anterior uveitis, ocular surface squamous neoplasia (OSSN), vascular tumors, and glaucoma [44,45]. Characterization and quantitative assessment of the ocular surface vasculature can assist in diagnosis, clinical grading of severity, and monitoring of response to treatments (Table 2). 

## 4. Optical Coherence Tomography Angiography of the Cornea

**Figure 1:**
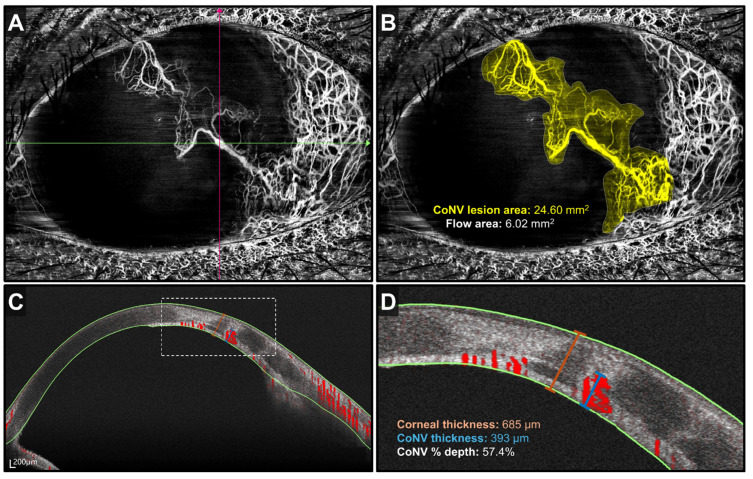
AS-OCTA scan of corneal neovascularization from traumatic corneal injury. (**A**) An en face image with whole blood flow signals. (**B**) Image with the total CoNV lesion area demarcated in yellow. (**C**) Cross-sectional scan along the green line on panel (**A**). Areas of vascularity are demarcated in red. (**D**) Close-up image of the dotted white box in panel (**C**). Image courtesy: Prof. Aijun Deng, Affiliated Hospital of Weifang Medical University, Shandong, China Device: BMizar, BM-400K, TowardPi Medical, China.

Currently, CoNV is clinically assessed via slit-lamp examination, which can be highly subjective depending on clinical experience. AS-OCTA has demonstrated the potential to detect and delineate CoNV in a rapid, non-invasive manner, allowing for the evaluation of vessel depth and density with good image quality and repeatability [40,46]. Early CoNV may not be detectable clinically on slit-lamp examination but was readily visualized on AS-OCTA, even in eyes with corneal opacification [47]. AS-OCTA is capable of visualization of deep CoNV, vessel depth measurement, and three-dimensional vasculature mapping, aiding clinicians in investigating the possible etiology of corneal injury (Figure 1). For example, interstitial keratitis secondary to herpes simplex virus would exhibit deep CoNV, whereas superficial or mid-stromal CoNV suggests varicella zoster virus [16,17]. Other than vessel depth, AS-OCTA also measures parameters such as vessel density (VD) and branch area. In addition to clinical assessment of CoNV severity and visual acuity [18,19], these AS-OCTA parameters have been shown to correlate well with contrast-based CoNV grading and leakage time [47]. This illustrates that AS-OCTA has the potential for adjunct usage in the clinical assessment of CoNV, as it is rapid and non-invasive, with results comparable or even superior to the current standard of contrast-based imaging.

CoNV is not only potentially sight-threatening but is also a significant risk factor for graft failure post-keratoplasty, as the cornea’s immunologically privileged state can be disrupted, increasing graft failure post-keratoplasty [6,21,48,49]. Pre-keratoplasty angioregressive treatment has therefore been explored to reduce the risk of graft failure in eyes with CoNV [50]. A diverse range of treatment options is available for CoNV, including topical steroids, cyclosporine, fine needle diathermy (FND), laser photocoagulation, and anti-VEGF therapy [21,50,51,52,53,54]. A reliable imaging modality to monitor the response to treatment is therefore essential for the optimization of CoNV treatment and management. Devarajan et al. compared the use of AS-OCTA and ICGA in rabbit models to monitor CoNV response to anti-VEGF treatment, and showed that both modalities were comparable in being able to detect CoNV regression [55]. Similarly, Foo et al. utilized AS-OCTA to evaluate treatment response to FND with anti-VEGF in human eyes with CoNV and corneal scarring, demonstrating that AS-OCTA can guide the pre-operative selection of vessels for FND as well as post-treatment monitoring [20]. In a study by Chan et al., CoNV was evaluated in eyes that had undergone penetrating keratoplasty (PK) or deep anterior lamellar keratoplasty (DALK) [21]. AS-OCTA was able to determine the depth of corneal vascularity post-keratoplasty. Hence, AS-OCTA can aid in the non-invasive assessment of CoNV, response to angioregressive treatment, as well as adding to the current imaging modalities available for post-operative monitoring of keratoplasty.

## 5. Optical Coherence Tomography Angiography of the Ocular Surface 

**Figure 2:**
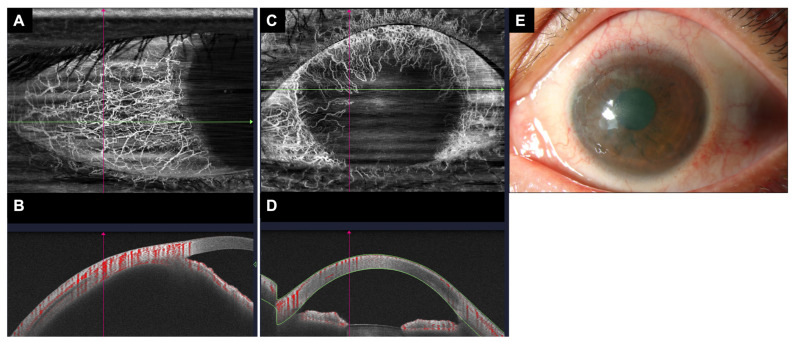
AS-OCTA scan of the conjunctival/sclera vasculature in a healthy eye versus an eye with limbal stem cell deficiency. (**A**) An en face image with whole blood flow signals in the healthy eye. (**B**) Cross-sectional scan along the green line on panel (**A**). Areas of vascularity are demarcated in red. (**C**) An en face image with whole blood flow signals in the eye with limbal stem cell deficiency. (**D**) Cross-sectional scan along the green line on panel (**C**) showing decreased areas of vascularity compared to the healthy eye in panel (**B**). (**E**) Slit-lamp photography image of the eye with limbal stem cell deficiency shown in panels (**C**,**D**).

AS-OCTA can also be used to assess limbal vasculature and its resultant impact on the ocular surface [56]. Limbal stem cell dysfunction can be associated with persistent corneal inflammation, abnormal vascularization, and corneal opacification with loss of visual acuity [22]. LSCD can arise from surgeries, topical ocular medications, Stevens–Johnson syndrome, and chemical injuries, among other causes [57,58,59]. Chemical ocular injury is one of the most common ophthalmic emergencies that can lead to LSCD [60,61], which is often preceded by ischemia of the limbus in the acute phase [23]. Chemical ocular injuries are largely evaluated at first presentation based on the Dua and Roper–Hall classifications, which determine the severity and prognosis of recovery [62]. These classifications could, however, be subjective and dependent on the clinician’s experience. Tey et al. showed that AS-OCTA can be used to assess the extent of limbal ischemia in the acute phase in rabbit models, and proposed a modified classification method using AS-OCTA [23]. The findings were similarly reproducible in human subjects, as demonstrated by Ang et al., demonstrating greater interrater agreement when assessing limbal disruption using AS-OCTA, as compared to slit-lamp examination (κ = 0.7 vs. κ = 0.4, respectively) [56]. Furthermore, Fung et al. also observed that limbal ischemia was more extensive when assessed using AS-OCTA compared to clinical examination, suggesting that AS-OCTA provides a more accurate and objective assessment of chemical ocular injury [63]. The use of AS-OCTA in the staging of primary LSCD has also been proposed by Binnoti et al., as the group found that two AS-OCTA derived parameters, namely, maximum corneal vascular extension and corneal vascular thickness, demonstrated good correlation with visual acuity and disease severity [22]. Therefore, AS-OCTA could potentially become an important adjunct imaging tool in providing an objective and reliable clinical assessment for chemical ocular injuries and LSCD (Figure 2), aiding clinicians in identifying, diagnosing, and grading the severity and prognosis of the disease.

AS-OCTA also has shown application in dry eye disease (DED). It was shown in a study by Yang et al. that conjunctival microvascular density was increased in patients with Sjogren’s disease and DED diagnoses [24]. AS-OCTA has also enabled insight into the interplay of inflammation, hypoxia, and angiogenesis in the development of DED [64]. In particular, the number of conjunctival vessels tends to increase with the severity of DED, and this could be explained by hypoxia and inflammation leading to activation of angiogenic factors and angiogenesis. Given that DED is a debilitating condition that requires more research, a greater understanding of the disease would enable timely diagnosis, better treatments, and outcomes in the long run. On the other hand, eyes with unstable tear films, as in DED, have been shown to potentially affect the quality of AS-OCTA images, as well as its repeatability [65], suggesting that more developments are required for AS-OCTA to contribute to clinical DED evaluation.

## 6. Optical Coherence Tomography Angiography of the Conjunctiva, Episclera, and Sclera 

AS-OCTA has been shown to provide a consistent quantitative assessment of ocular surface vasculature in both healthy and diseased eyes [66]. It can be employed to visualize and characterize the vasculature of various ocular surface lesions such as OSSN, melanocytic lesions, and other benign lesions [45,67]. OSSN is a heterogeneous group of pathologies of the ocular surface epithelium, ranging from cornea and conjunctiva intraepithelial neoplasia to carcinoma in situ and invasive squamous cell carcinoma. Clinical differentiation of OSSN can be challenging due to similar presentations, and the thickness of the lesion is not always indicative of a more invasive or severe pathology [68]. The advent of AS-OCTA has shed insight into angiogenesis in OSSN, such as the breakdown of normal conjunctival vasculature [26]. Liu et al. demonstrated that AS-OCTA could delineate the vascular network of an OSSN with its surrounding structure. Specifically, a greater vessel area density (VAD) in the subepithelial tissue and the tissue underneath the conjunctival component of the tumor was observed, compared to an unaffected eye [45]. When comparing the vasculature of benign and malignant lesions, there seemed to be marked morphological and quantitative differences between both lesions when seen on AS-OCTA—malignant lesions tend to have a greater diameter and peri-lesional vessel depth and diameter, which may represent feeder vessels [25]. Treatment response in OSSN can also be observed and monitored with AS-OCTA. Theotoka et al. conducted a study on OSSN treated with topical immunotherapy or chemotherapy, which revealed a significant decrease in subepithelial VAD during treatment and final VAD, comparable to the non-affected eye with tumor resolution [27]. Tumor vascular density derived from AS-OCTA could potentially be used in future criteria for malignancy grading [60].

OSSN can sometimes be difficult to differentiate clinically from other benign lesions such as pterygium, especially in the early stages of disease or history of ocular surgery [26]. AS-OCTA could potentially be used to aid the differentiation between OSSN and other ocular surface lesions such as pterygium and melanoma [69]. Nampei et al. found and described the different flow patterns in OSSN and pterygium on AS-OCTA—“zigzag vessels” in both the superficial and deep layers in OSSN, but “straight vessels” in the superficial layer in pterygium [26]. Kiseleva et al. also demonstrated AS-OCTA’s ability to differentiate conjunctival melanoma and nevus based on conjunctival perfusion density (PD)—conjunctival PD was significantly higher in conjunctival melanoma when compared to nevus [70].

AS-OCTA has also recently been utilized in the management of pterygium. The reperfusion of conjunctival autografts (CAGs) in eyes after pterygium removal surgery was evaluated via AS-OCTA, demonstrating an inverse correlation between CAG thickness and revascularization density [28,29]. AS-OCTA, hence, could be utilized for post-operative monitoring of autograft survival in pterygium surgery. 

AS-OCTA is also beneficial in evaluating pathologies of the sclera and episclera, most commonly scleritis and episcleritis. Scleritis affects the superficial and deep episcleral capillary plexus, whereas episcleritis only affects the superficial episcleral capillary plexus [30]. Routine clinical examination can usually differentiate between the two conditions: the vascular engorgement in scleritis produces a characteristic bluish-violet hue while a distinct red hue can be seen in episcleritis [71]. The use of topical phenylephrine could also be used to clinically distinguish between the two entities [72]. However, these signs can be subjective and subtle. Furthermore, the clinical symptoms of globe tenderness and pain, typically indicating scleritis, can be confounded with the use of analgesia. While episcleritis itself is often benign, scleritis can be a harbinger of something sinister such as systemic vasculitis like systemic lupus erythematosus or polyarteritis nodosa [73,74]. Timely diagnosis is important, as late treatment could lead to scleral thinning, perforation, melting, and ultimately permanent blindness [75]. Specific patterns on FA and ICGA such as leakage scores have been shown to help discriminate episcleritis and scleritis, but are often limited due to their invasive nature and associated adverse effects of contrast use [76,77]. Studies have demonstrated the added utility of AS-OCTA in diagnosing and distinguishing between episcleritis and scleritis. For example, a study by Hau et al. revealed a significant increase in overall vessel density index (VDI) in the episclera–sclera complex of eyes with scleritis and episcleritis compared to healthy eyes, as well as greater VDI in scleritis compared to episcleritis [30]. This highlights potential quantitative markers derived from AS-OCTA that can aid in the diagnostic workup and management for both conditions. Another longitudinal study also revealed an AS-OCTA-derived parameter, scleral area VD, which was shown to directly correlate with the severity of anterior scleritis [31]. This parameter can potentially enable the clinical and objective quantification of scleral inflammation, allowing for identification and further workup for eyes that are refractory to initial scleritis treatment.

AS-OCTA also provides a non-invasive and less time-consuming alternative that can quantitively evaluate the intrascleral and conjunctival vessels implicated in glaucoma, at varying depths and locations [10]. Akagi et al. discussed the role of AS-OCTA images in objectively assessing conjunctival hyperemia in treated glaucoma eyes [78]. An extension to the study has been applied to trabecular bypass minimally invasive glaucoma surgery (MIGS), where episcleral VD was found to decrease post-operatively, which can be attributed to increased aqueous outflow within the episcleral veins and hence reduced signal intensity detected by AS-OCTA [79]. A separate study by Okamoto et al. also found that lower intrascleral VD corresponded to greater surgical success rate and intraocular pressure (IOP) reduction post-MIGS [32]. In trabeculectomies, the success of IOP reduction is dependent on the function of conjunctival blebs [78]. A study by Hayek et al. found lower IOP post-trabeculectomy in conjunctival blebs that were hypovascularized compared to those that were hypervascularized, as determined by AS-OCTA [31]. Less vascularized blebs were also associated with less inflammation and a lower risk of fibrosis and bleb failure. AS-OCTA was also able to measure and quantify microcyst density in blebs, which showed an inverse correlation with IOP [31]. Given that the signs of early bleb failure are subtle, early detection and intervention could be difficult without objective assessment. There is potential for AS-OCTA to be incorporated in routine assessment pre- and post-trabeculectomy to provide an objective analysis of the risk of bleb failure.

## 7. Limitations and Future Development

OCTA systems were initially introduced for evaluation of the posterior segment of the eye. While the research and implementation of OCTA for the posterior segment are fairly robust, adaptation for the anterior segment vasculature is still a relatively recent development with its own set of limitations. Firstly, various sources of image artefacts can affect the analyses of AS-OCTA of the cornea and ocular surface. For example, OCTA systems that are employed for the anterior segment are unassumingly posterior segment OCTA systems with the addition of an anterior segment adaptor lens and modified scanning protocols [1,80]. Hence, image analysis software that is inherently built for posterior segment analyses may lead to non-parallel segmentation and artefacts caused by light scatter due to corneal refraction, causing imprecise vasculature density calculations during depth-resolved analyses [4]. Motion artefacts secondary to saccadic eye movements are prevalent in AS-OCTA imaging, as motion correction systems have yet to be designed or implemented, leading to poorer image quality [81]. Projection artefacts on the deeper vasculature layers caused by more superficial vessels due to light scattering can inadvertently cause misinterpretation as abnormal or additional vessels by image analysis software, thereby affecting vasculature density computation [81]. AS-OCTA currently also lacks tracking capabilities for comparing serial scans in the same precise location.

These can be, however, circumvented by performing and comparing multiple scans or correlation with other imaging modalities such as slit-lamp photography [82]. Furthermore, advancements in machine learning and artificial intelligence can produce analysis software with superior auto-segmentation capabilities to reduce image artefacts [83]. The area examined by AS-OCTA has also been fairly limited when compared to its posterior segment counterpart, which, although initially limited, was expanded when wide-field OCTA (using a montage technique) was introduced. This could similarly be applied in the anterior segment, which would enable greater clarity of the examined area.

## 8. Conclusions

AS-OCTA is an emerging imaging modality that permits a rapid, non-invasive assessment of the vasculature of the anterior segment. Although initially designed for posterior segment evaluation, there has been an increasing number of studies on the clinical translation of AS-OCTA for the diagnosis and management of ocular pathologies. These include anterior segment vascular lesions and tumors, ocular surface diseases, and prognostication of graft rejection post-keratoplasty [4,84]. The en face imaging of AS-OCTA in multiple coronal planes provides an intuitive perspective on the anterior segment vasculature that clinicians can directly correlate with their observations on a clinical slit-lamp examination. While still in its infancy, further optimization of AS-OCTA and image processing software, along with the integration of artificial intelligence, presents a foreseeable future for its use in clinical practice.

## Figures and Tables

**Table 1:** Advantages and disadvantages of anterior segment optical coherence tomography (AS-OCT) angiography vs. traditional dye-based angiography.

	Advantages	Disadvantages
AS-OCT angiography	1. Non-invasive and relatively time-efficient 2. Zero risks of contrast-related adverse effects 3. Does not require clinician to operate	1. Image quality can be affected due to image, motion and projection artefacts 2. Operator-dependent
Traditional dye-based angiography (fluorescein angiography and indocyanine green angiography)	1. Ability to differentiate normal and abnormal vessels even in corneal scarring 2. Contrast leakage helps differentiate afferent and efferent vessels while providing details about vessel maturity and pathology	1. Contrast leakage can obscure visualization of deeper vessels 2. Invasive and time-consuming 3. Risks of contrast-related adverse effects 4. Limited use in hepatic and renal impairments 5. Requires clinician to perform 6. Operator-dependent

**Table 2:** Potential applications of anterior segment optical coherence tomography angiography in the cornea and ocular surface.

Anatomical Location	Pathologies	Potential Applications
Cornea	Corneal neovascularization (CoNV)	Diagnosis: superficial or mid-stromal CoNV in interstitial keratitis is suggestive of varicella zoster virus while deep CoNV suggests herpes simplex virus [16,17] Assessment: correlation of vessel density to CoNV severity and visual acuity [18,19] Assessment/treatment response: pre-operative selection of vessels for FND as well as post-treatment monitoring response to FND with anti-VEGF in human eyes with CoNV and corneal scarring [20] Treatment response: depth of corneal vascularity post-PK and DALK [21]
Ocular surface	Limbal stem cell deficiency	Assessment: objective staging of limbal ischemia [22,23]
	Dry eye disease	Assessment: positive correlation between number of conjunctival vessels and severity of disease [24]
Conjunctiva, episclera, and Sclera	Ocular surface squamous neoplasia (OSSN)	Diagnosis: greater diameter and peri-lesional vessel depth and diameter in malignant lesions compared to benign lesions [25] Diagnosis: differentiation from other lesions such as pterygium (“zigzag vessels” in both the superficial and deep layers in OSSN and “straight vessels” in the superficial layer in pterygium) [26] Treatment response: decreased subepithelial vessel area density post-treatment with topical immunotherapy or chemotherapy [27]
	Pterygium and conjunctival autografts	Treatment response: inverse correlation between post-operative thickness and revascularization of autograft [28,29]
	Episcleritis and scleritis	Diagnosis: greater vessel density index in scleritis compared to episcleritis [30] Assessment: positive correlation between vessel density and severity of scleritis [31]
	Glaucoma	Treatment response: decreased episcleral vessel density post-MIGS [32] Treatment response: lower IOP in hypovascularized conjunctival blebs compared to hypervascularized blebs post-trabeculectomy [31]

DALK—deep anterior lamellar keratoplasty; FND—fine needle diathermy; IOP—intraocular pressure; MIGS—minimally invasive glaucoma surgery; PK—penetrating keratoplasty.
